# Warner against Griffin

**Published:** 1857-06

**Authors:** 


### WARNER AGAINST GRIFFIN.

U. S. CIRCUIT COURT, NOVEMBER 14, 1856.—JUDGE NELSON'S CHARGE.

Gentlemen, the patentee in this case presents a claim for the discovery, first of the cement or compound described in his specification, which is used in constructing the gums of teeth; and in the second place, the process of setting teeth by means of this cement, thereby making a continuous gum in connection with the teeth. Now, your attention must first be called to the construction of this patent, with a view of ascertaining the exact thing which the patentee has discovered and has patented, and for the protection and enjoyment of which discovery or improvement, against alleged infringers, this suit has been brought.

The patentee states in his specification, that "the cement may be formed of any of the known fluxes, combined with silex, wedge-wood and asbestos,"—those three articles intermixed with gold and platina scraps; and then he goes on and gives the proportions of the three articles, and of the materials used as fluxes, for the purpose of fusing the mass. Any fluxes may be used. There is nothing in the patent, as it respects this portion—that is, the fluxes used for the purpose of fusing the other three ingredients, presenting any claim to those articles. They may be taken at large, and at the discretion and judgment of the artist or the manufacturer, but they are to be combined with silex, wedge-wood and asbestos, in the proportions which are stated in the patent, and which is the formula that the patentee prefers in the manufacture of cement: that is, silex 2 oz.; wedge-wood 1½ oz.; asbestos 2 drms.; and then he gives flint-glass, borax, feldspar, and kaolin clay as the fluxes. Now, the cement is manufactured out of these ingredients, according to the directions given by the patentee. And then we have the *process*. He says: "I construct my plates, and arrange the teeth thereon in the usual way. I then apply the cement in a plastic state upon the outside, between and around the base of the teeth, so as to form an artificial gum upon the teeth and plate. The teeth and gum are then covered with a mixture of asbestos and plaster of Paris, mixed with water and reduced to a plastic state. The teeth being thus covered, the wax is removed from the inside of the teeth, and the cement is applied thereon, and also upon the plate, so as to fill up all the interstices around the base of the teeth. When the cement and mixture thus applied have become thoroughly dry, the work, so united, is put into a furnace sufficiently heated to fuse the cement, and immediately after the fusion thereof, it is withdrawn from the furnace and cooled slowly. The plaster mixture is then removed, and a gum color applied."

Now this process, which I have called your attention to, of constructing the gum in connection with the teeth, is embraced in his first claim, in the following words:

"What I claim as my invention, and desire to secure by letters patent, is a new mode of setting mineral teeth on metallic plate, by means of a fusible silicious cement, which forms an artificial gum, and which also unites single teeth to each other and to the plates upon which they are set."

I ought to have called your attention previously to another clause. After the gum color is applied, he says:

"The work is again placed in the furnace as before, and when fused is withdrawn and cooled as before, by which means the metallic back plates, solder, and blow-pipe are dispensed with, although back plates may be attached to the teeth, if desired."

Now with respect to this process, which is the first claim, according to the true construction of the specification, the patentee claims to have invented a compound, by which, in pursuing the process which he has described in setting the teeth, and forming a continuous gum, both on the inside and outside,—he claims that by this discovery of a compound, and the process of applying it, in constructing the gum in connection with the teeth, that he can thereby dispense with the back plates and soldering which were used before, although back plates may be used, if desired.

I do not agree with the learned counsel for the plaintiff, that upon a true interpretation of this specification, the patentee claims to embrace within his discovery or improvement in the manufacture of teeth, the use of this cement, including the back plates or fastenings of the teeth, according to the previous mode; but on the contrary, according to my view of the patent, he claims to have invented an article and a process of using it, by which these back plates may be dispensed with, and the cement and the process will constitute a complete, practical, and useful article. That back plates may be used, if desired, is merely a suggestion outside of this invention. It may be used as it was used before,—may be added to the invented article, by using it in the construction of the teeth; but this is outside of the thing claimed to have been invented; and unless, according to my view of the patent, the cement and the process described by the patentee, excluding the back plates and fastenings, constitutes a practical and useful article, the invention is not the subject of a patent. Because the object of the law, in requiring the patentee to describe his invention, is that persons skilled in that art or branch of knowledge may construct or manufacture the article by pursuing his description; and it is quite plain that the instruction which is given for the benefit of the public, in the manufacture of that article, would lead the manufacturer to make it, excluding or dispensing with the back fastenings. The others may be used if the manufacturer desires it, but they are not necessary, they are not material, they may be left out or dispensed with; and therefore the article manufactured by the process described by the patentee, aside from and independent of these fastenings, by means of back plates, must be a practical article, and useful for the purpose for which it is described and constructed.

Now as respects the degree of utility, that is another question. If the article, thus constructed, dispensing with the back fastenings, is not the very best article of the kind, why that fact or that view will not invalidate the patent. If the article is better by adding the back plates than it would be by dispensing with them, that is not a reason for invalidating the patent. The validity of the patent does not depend upon its degree of utility, as compared with other articles of a similar description. It may not be as good, yet if it be new, it is patentable, and the inventor is entitled to the benefit, and it is no ground of defense to set up on the part of the infringer, that, although he uses it, yet there is a better article used, which he might have used. That is not an answer. Although it may be inferior in degree, and so little in demand that the patentee may not derive any great benefit from it, yet if it is new and practicable, it is a patentable subject, and he is entitled to be protected. But then it must be an article so useful, that when devoted to the purposes for which it was discovered and applied by the inventor, it will be practically beneficial, —reasonably useful,—in order to constitute the invention a patentable subject.

Now, upon the part of the learned counsel for the defendant, it has been insisted, and testimony has been produced, in the progress of the trial, to show that an article made according to the specification, both as to the compound and to the process, if followed strictly, dispensing with the back plates, is a failure—is not practicable, and can not be used successfully.

On the part of the learned counsel for the plaintiff, they have introduced testimony for the purpose of refuting this ground of defense—they maintaining that if constructed precisely according to the directions in the specification, dispensing with these back plates, it is still a useful article, still practical, and the proper subject of a patent.

Now I am not going to occupy your time in going over this testimony. I have no doubt it is fresh in your recollection, and you will be able to recall the material witnesses and facts upon this question, and come to an intelligible determination. It is a question of fact—one which belongs exclusively to the Jury to determine.

It is also set up as another ground of defense, that this article is not new, and that therefore the patent is invalid. Now as it respects the process of manufacturing the article, in order to determine whether it is new or not, you must take into consideration the compound or cement as described by the patentee, because it is that article, that specific cement, which he claims to have discovered, and which he uses in the process of constructing artificial teeth; and in your inquiry whether or not the article is new or old, you must keep in your minds the question whether a cement substantially like that described by the patentee has been used heretofore in the construction of artificial teeth, and not whether artificial teeth may not have been constructed after the same process—that is, by setting up the teeth upon a plate, and by putting around it a continuous gum of cement, of some description, and baking or hardening it. That is not the inquiry; but the inquiry is, whether previous to the invention of Dr. Allen, artificial teeth had been constructed according to this process, which process is not new, but a substantially similar cement, or a similar compound. That is the question. And hence, even if you should come to the conclusion that teeth had been formed according to this process, previous to the invention of Dr. Allen, by any sort of compound or cement, that would not be an answer to the action; because that is not what is claimed as the thing discovered or invented. That is, first, the cement as described in the patent, or the compound described and used according to this process in constructing the article. You must not only take into consideration the process, which may not be new, but also the character of the cement which is used in the construction of the article.

Now you have a great deal of testimony that has been introduced, both on the part of the plaintiff and of the defendant, bearing on this question. I am not going over it. It will be for you, after a proper consideration of the testimony, to determine for yourselves whether or not this cement and process was new or not at the time the plaintiff claims to have made the improvement.

Now there is another question in the case that has been litigated and contested throughout the trial, and which it will be necessary for you to take up and determine. It is insisted upon the part of the defendant, that the proportions which are specified by Dr. Allen, in making his cement, will not, if strictly followed, create or be the means of producing a practical, useful article. You have heard the witnesses on both sides of this question. Experiments have been made on both sides. It is alleged on one side that if the patent is followed to the letter, the article manufactured according to the description will be useless—that it will not be practicable—that it will be rather an imposition on the public. Upon the part of the plaintiff it is claimed that, on the contrary, it will produce a useful, practical article. Now all I have to say to yon upon this branch of the case is this: that you must be satisfied—(because the patentee assumes this burthen)—that by following the description according to the letter of the patent, a useful and practical article can be manufactured by a skillful person, or one of competent skill; otherwise the subject is not patentable; because, to sustain the patent, a manufacturer who, after it has expired, and the public are entitled to the benefits of the discovery, shall follow the description and directions to the letter, must be enabled to make a useful and practical article, or else the patent is invalid. I know it was said by the plaintiff's counsel that there might be slight variations, and that those slight variations would not operate to invalidate the patent. I agree to that, so far as it regards the question of infringement. I agree that *there* a slight variation of the proportions of the articles by an infringer would not be a ground for evading the patent; but when the question is whether the article manufactured according to the patent is a useful or practical article, it must be so in a case where the manufacturer has followed the proportions according to the description, to the letter; and if it fails after that process has been adhered to or followed, the patent can not be upheld.

Now these are all the observations I shall make to you on the first claim. The second claim is as follows: "I also claim to be the inventor of said cement or compound, a full and exact description of which is herein given."

Now that depends upon the question whether this compound, as described, and its proportions, is new, or whether it is old. That is a compound of silex, wedge-wood, and asbestos, in the proportions set out in the patent, with any of the usual fluxes. If that is a new compound—a new cement never before having been manufactured, it undoubtedly constitutes a patentable subject. Otherwise not. It was said by the counsel for the defendant that this claim was defective, upon the ground that the claim was not for the art of manufacturing a compound, as distinguished from the article itself after being thus compounded. Well, that would be so upon this condition or view of the case: if this mixture of the three ingredients with any of the known fluxes had been before made and was known, and the patentee had taken up that old mixture, and by changing the proportions of the same ingredients had made a different article—an article of a different character, then the patentee must claim for the art of composing the compound, and not for the compound itself; because upon the hypothesis I stated, that was an old article. But in this case the patentee claims the compound itself as a new article, never before mixed in the way he has described it. But it must be remarked in connection with this second claim, that this article or compound, formed into cement according to the description of the patentee, when used in the process claimed under the first claim in the manufacture of teeth, according to the mode described by the patentee, must produce a practical, useful article—because the utility of this cement depends upon the use of it in the process of manufacturing artificial teeth; and if, when used in the way for which it was combined, it was not successful, then it will fail for the want of utility. But if, on the other hand, when used according to the description in the specification, a useful article is produced, then, in my apprehension, the second claim would be maintained of the patent.

Now I have very nearly finished what I intend to say in this case. You will first turn your attention to the question whether or not teeth, manufactured according to the directions of the patent, laying out of view back plates or fastenings, is or is not a useful, practical article. If it is, then the plaintiff, so far as this first claim is concerned, I think has sustained his patent. If, on the other hand, you come to the conclusion that without the intervention of the back straps and solder, teeth manufactured according to the description in the patent would be impracticable and useless, then I think the defense has been established.

In the next place, if the plaintiff fails upon the first ground —that is, upon the process for constructing the artificial teeth, then I think he fails upon the second claim, because it involves the inutility of the cement. If the plaintiff, in your judgment, has maintained the claim, on the construction I have given it, on the first ground, then I think he has on the second, because the first ground involves the utility of the cement, which is involved in the second claim.

The next question, supposing you shall come to the conclusion, on the evidence, that the patent is valid, will be whether or not the defendant has infringed.

Now, I agree with the counsel for the defendant, that if it appeared in this case that the defendant had purchased the cement of Allen, at one of the dental depots, from a person licensed by the patentee, or his assignee, to sell the cement for the purpose of manufacturing teeth, that that of itself would be a defense, so far as it respects the second claim, which is simply for the compound or cement: I agree that a sale under the authority of the patentee would be an answer to that claim, because if he puts his cement upon sale, why, any body who buys it is not responsible simply for the compound. But in this case the weight of evidence seems to be that the article purchased and used by the defendant, in the manufacture of the teeth for Mrs. Davis, was an article on sale, manufactured from the formula of Dr. Hunter, and therefore, though on sale by a person keeping one of the dental depots, yet he had no authority from the patentee or assignee to sell the article, therefore this ground of defense, I apprehend, is not available.

The only question, it seems to me, involved in the point of infringement, is this: whether or not the formula of Dr. Hunter is or is not substantially the same as the formula of the patentee. If it is substantially different, then the use of it is not an infringement; if it is substantially the same, then it would be.

Now it is said that the formula of the patentee, taken strictly from the patent, would not produce, in process of manufacture, a substantial or useful article; and they argue further that the proportions have been changed, since the patent, by the patentee himself, and also by his assignees and agents, and that it is in consequence of this change of the proportions, this variation from the formula of the patent, that the party has obtained an article which is available for the purpose of making artificial teeth. Now all I mean to say to you upon this subject is this: I have said, and I repeat it, that you must be satisfied in the first place, that the formula, as found in the patent, is sufficient for the purpose of making a useful article, and if you come to that conclusion, then any unimportant variations, by way of difference in proportions, formal variations, or changes in proportion, would be an evasion of the patent. The patent is not to be evaded in that way, by formal unimportant variations, by changing the proportions; because some latitude may be indulged in this respect, without changing the original character of the article; and hence in that view these changes, unless radical, would not constitute a defense, although made in the formula of Dr. Hunter, used by the defendant.

But if you come to the conclusion that this change of proportion is essential for the purpose of making a useful article, and that without it a useful article can not be made, then undoubtedly if that is the character of the change in the formula of Dr. Hunter, it would be outside of the other, would be a different article, and would not be an infringement.

These are all the observations I deem it necessary to make. On the question of damages, if you come to that, the plaintiff only asks six cents.

A Juror: If we conclude from the evidence that Mr. Hunter uses and sells his compound by permission of Dr. Allen, would the defendant be guilty of infringement?

The Court: There is no evidence of that kind in the case.

Verdict for the defendant.—*Dental Recorder.*

